# Prognostic Prediction Models for Ulcerative Colitis: Systematic Review and Meta-Analysis

**DOI:** 10.2196/71944

**Published:** 2025-12-22

**Authors:** Zhijun Bu, Yuan Sun, Zeyang Shi, Liming Hu, Chuanlan Ju, Yuan Liu, Jing An, Huiyi Sun, Jianping Liu, Zhaolan Liu

**Affiliations:** 1 School of Traditional Chinese Medicine, Centre for Evidence-Based Chinese Medicine, Beijing University of Chinese Medicine, 11 Beisanhuan East Road, Chaoyang District, Beijing, 100029, China, 86 17800176627; 2 Department of Spleen, Stomach, Liver & Gallbladder Diseases, Dongfang Hospital, Beijing University of Chinese Medicine, Beijing, China; 3 Department of Public Health, Yantai Municipal Hospital of Traditional Chinese Medicine, Shandong, China; 4 Department of Spleen and Stomach Diseases, Third Affiliated Hospital, Beijing University of Chinese Medicine, Beijing, China; 5 Department of Spleen and Stomach Diseases, Dongzhimen Hospital, Beijing University of Chinese Medicine, Beijing, China

**Keywords:** ulcerative colitis, prognostic prediction models, Prediction Model Risk of Bias Assessment Tool, PROBAST, logistic regression, systematic review and meta-analysis

## Abstract

**Background:**

Ulcerative colitis (UC) is a chronic inflammatory disease with highly variable symptoms and severity. Prognostic models for UC support precision medicine by enabling personalized treatment strategies. However, the quality and clinical utility of these models remain inadequately assessed.

**Objective:**

This study aimed to systematically review and critically evaluate the development, performance, and applicability of prognostic prediction models for UC.

**Methods:**

To identify prognostic models for UC, a comprehensive search was conducted in PubMed, Embase, the Cochrane Library, Web of Science, SinoMed, China National Knowledge Infrastructure, Wanfang, and VIP Database up to November 2, 2024. Extracted data included study characteristics, model development methods, validation metrics (eg, area under the curve and concordance index). The risk of bias and applicability were evaluated using the Prediction Model Risk of Bias Assessment Tool. A meta-analysis was conducted to assess model performance.

**Results:**

A total of 30 studies involving 7452 patients with UC were included, with the largest numbers conducted in China (11/30, 37%) and Japan (4/30, 13%). Most studies were retrospective (22/30, 73%). The primary objectives of the UC prognostic models included predicting therapeutic effects and responses to treatment, particularly to tumor necrosis factor-alpha inhibitors (eg, infliximab and adalimumab), and assessing the risks of surgery, disease progression, or relapse. Logistic regression was the most frequently used method for both predictor selection (6/30, 20%) and model construction (12/30, 40%). Common predictors included age, C-reactive protein, albumin, hemoglobin, disease extent, and Mayo scores. The meta-analysis yielded a pooled area under the curve of 0.84 (95% CI 0.77‐0.92). Most studies exhibited a high risk of bias (29/30, 97%), particularly in participant selection and statistical analysis. Applicability concerns were identified in 18 studies (18/30, 60%), primarily due to subgroup-specific designs that limited the generalizability of the findings. External validation data (14/30, 47%) were limited, and only a small number of studies (12/30, 40%) included calibration curves or decision curve analysis.

**Conclusions:**

This study demonstrates that prognostic models for UC have some potential in predictive performance and clinical application. However, most models are constrained by high bias risk, insufficient external validation, and limited generalizability due to small sample sizes and subgroup-specific designs. Future research should prioritize multicenter validations, refine model development approaches, and enhance model applicability to support broader clinical implementation.

## Introduction

Ulcerative colitis (UC) is a chronic inflammatory bowel disease involving the rectum and colon, characterized by a relapsing-remitting course and affecting individuals across all age groups [1,2]. While UC incidence has stabilized in early industrialized regions, it is rising rapidly in newly industrialized countries, especially in Asia and Latin America [3]. A recent global analysis of over 500 population-based studies proposed a 4-stage model of inflammatory bowel disease evolution and classified many countries, including China, Malaysia, and Brazil, as being in the *accelerating incidence* stage, with steadily increasing UC burden [4]. In China, UC has transitioned from being a rare condition to a prevalent one, accounting for up to one-quarter of inpatient beds in gastroenterology and colorectal surgery departments [5]. The underlying mechanisms of UC are complex and involve a combination of genetic susceptibility, epithelial barrier impairments, immune system disturbances, and environmental triggers [6]. The disease impairs quality of life because of its relapsing nature and treatment burden, affecting mental health and social functioning [7].

UC exhibits substantial heterogeneity in symptoms and the severity of inflammation among patients. Consequently, relying solely on symptom-based approaches to guide treatment often leads to suboptimal management [8]. Precision medicine focuses on creating customized treatment strategies based on the unique characteristics of each patient and the progression of their disease, playing a crucial role in enhancing therapeutic effectiveness. By identifying each patient’s specific response to treatment, unnecessary medication use can be avoided, side effects minimized, and treatment efficacy optimized [9,10]. Prognostic prediction models are central to precision medicine because they allow clinicians to predict disease progression, treatment response, and survival, thereby enabling more individualized treatment strategies [11]. However, despite the growing number of UC prognostic models, comprehensive evaluations of their methodological quality, external validity, and real-world clinical utility are still lacking.

This study aimed to systematically review and summarize the existing literature on UC prognostic prediction models; comprehensively assess their performance, external validation, and clinical utility; identify existing research gaps; and provide evidence to guide the future development of UC prognostic prediction models.

## Methods

### Overview

This study was registered in the International Prospective Register of Systematic Reviews under the registration number CRD42024609424. It adheres to the 2020 PRISMA (Preferred Reporting Items for Systematic Reviews and Meta-Analyses) guidelines [12], and the full 2020 PRISMA checklist is provided in Checklist 1.

### Search Strategy

A systematic search was conducted across 8 databases: PubMed, Embase, Cochrane Library, Web of Science, SinoMed, China National Knowledge Infrastructure, Wanfang Database, and VIP Database. The search covered the inception of each database up to November 2, 2024. Boolean operators (AND and OR) were used to combine Medical Subject Headings and free-text keywords, and detailed search strategies for all databases are provided in Multimedia Appendix 1.

### Inclusion and Exclusion Criteria

The inclusion and exclusion criteria were systematically structured based on the population, intervention, comparator, outcome, and study design (PICOS) framework, with additional relevant domains (eg, language) incorporated, as summarized in Textbox 1.

Textbox 1. **Inclusion and exclusion criteria categorized by eligibility domain, based on the population, intervention, comparator, outcome, and study design (PICOS) framework with additional language criteria.**

Inclusion criteria

- Population: adult patients diagnosed with ulcerative colitis (UC)
- Intervention: development and validation of prognostic prediction models
- Comparator: not applicable
- Outcome: clinical outcomes, such as relapse, colectomy, hospitalization, steroid dependence, and so on
- Study design: original studies using statistical or machine learning models for prognosis
- Language: English or Chinese

Exclusion criteria

- Population: studies involving non-UC populations (eg, Crohn disease and inflammatory bowel disease without UC-specific data)
- Intervention: studies focusing solely on diagnostic models or without a clear time lag between predictors and outcomes
- Comparator: not applicable
- Outcome: studies not reporting prognostic outcomes or focusing only on diagnostic classification
- Study design: reviews, systematic reviews, case reports, editorials, or studies without predictive model development or validation
- Language: languages other than English or Chinese

### Study Selection and Screening Process

InInitially, 1 researcher (ZB) imported all retrieved records into EndNote X9 (Clarivate) software for deduplication. The deduplicated records were independently reviewed by 2 researchers (YS and ZS) through titles, abstracts, and keywords to identify studies that met the inclusion criteria. The same researchers reviewed the full texts of potentially eligible studies to finalize inclusion based on the criteria. At each stage, another researcher (LH) cross-checked the results to ensure consistency. Discrepancies were resolved through discussions involving all 3 researchers until a consensus was reached.

### Data Extraction

Data from the included studies were independently extracted by 2 researchers (YS and ZS) and documented in a Microsoft Excel spreadsheet. Data extraction and organization were performed using Microsoft Excel (version 2024). Extracted data included basic study information such as the title, first author, publication year, study region, and so on.

1. Study background: specific subgroups of patients with UC (eg, mild, moderate, or severe cases)
2. Study population: sample sizes of training, validation, and test datasets; single-center or multicenter studies; and so on
3. Prediction model development: number and types of predictor variables, methods for variable selection, modeling approaches, data partitioning methods, and use of cross-validation or bootstrap validation
4. Model performance: metrics for internal and external validation, such as the area under the curve (AUC) and concordance index (C-index)
5. Applicability and limitations: clinical application scenarios and study limitations

Another researcher (CJ) cross-checked the extracted data with the original reviewers. Disagreements were addressed through discussion among the 3 researchers until mutual agreement was achieved.

### Quality Assessment of Included Studies

Two researchers (YL and JA) independently evaluated the quality of the included studies using the Prediction Model Risk of Bias Assessment Tool (PROBAST). This tool assesses bias risk and applicability in prediction model studies across 4 domains: participants, predictors, outcomes, and analysis. It also includes 20 signaling questions designed to pinpoint potential biases in study design, data analysis, and reporting. Additionally, PROBAST assesses the applicability of models within specific clinical contexts. Each study’s risk of bias was categorized as “high risk,” “low risk,” or “unclear risk” [13]. Differences were addressed through discussion involving a third researcher (HS).

### Data Synthesis and Statistical Analysis

The synthesis of data and statistical analysis were conducted using Stata 17 (StataCorp LLC) software. Meta-analyses of performance metrics were conducted using the meta package and the metagen function, with subgroup analyses comparing internal and external validation datasets. A random effects model was used to estimate pooled effect sizes with 95% CI, and heterogeneity was evaluated using conventional metrics. Funnel plots and the Egger test were used to assess potential publication bias. Sensitivity analyses were performed by excluding individual studies one at a time to evaluate the stability of the results and the impact of each study on the pooled effect sizes.

## Results

### Study Selection

**Figure 1.**
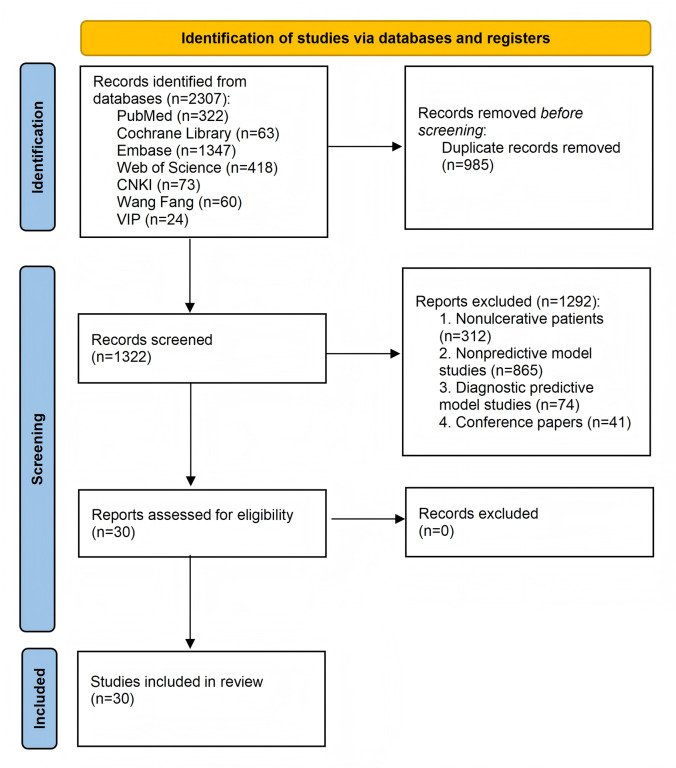
Study selection flow diagram based on PRISMA (Preferred Reporting Items for Systematic Reviews and Meta-Analyses) 2020 guidelines for a systematic review and meta-analysis of prognostic prediction models in ulcerative colitis (UC). This figure illustrates the number of records identified, screened, excluded (with reasons), and included in the final analysis. The systematic review included studies that developed or validated prognostic models for patients with UC, published in either English or Chinese, from the inception of each database up to November 2, 2024, across various countries. The PRISMA 2020 framework was used to ensure transparency and reproducibility in the study selection process.

A total of 2307 studies were identified from the databases. After removing 985 duplicates (985/2307, 42.70%), 1322 studies (1322/2307, 57.30%) remained for screening. During the preliminary screening, we excluded 312 studies (312/1322, 23.6%) that did not include patients with UC, 865 studies (865/1322, 65.4%) unrelated to prediction models, 74 studies (74/1322, 5.6%) focusing on diagnostic prediction models, and 41 (41/1322, 3.1%) studies focusing on conference abstracts. Consequently, 30 studies (30/1322, 2.3%) were chosen for a full-text review. After independent verification and discussion, all 30 studies were ultimately included in the analysis (Figure 1).

### Study Characteristics

The studies included in the analysis were published between 2015 and 2024, involving a total of 7452 patients with UC. Among these, 12 studies (12/30, 40%) focused on patients with moderate-to-severe UC, and 6 (6/30, 20%) exclusively targeted patients with severe UC. Geographically, the largest number of studies was conducted in China (11/30, 37%), followed by Japan (4/30, 13%). With respect to study design, 6 studies (6/30, 20%) were prospective, 22 (22/30, 73%) were retrospective, and 2 (2/30, 7%) used a mixed prospective-retrospective approach. Ten were single-center studies (10/30, 33%), whereas 20 involved multiple centers (20/30, 67%).

Key objectives of the included UC prognostic models included predicting treatment efficacy and response, assessing surgery-related risks, forecasting disease progression or relapse, and exploring molecular biomarkers or disease mechanisms. Treatment efficacy and response prediction was the most common objective (18/30, 60%), focusing on interventions such as tumor necrosis factor-alpha (TNF-α) inhibitors (eg, infliximab and adalimumab), vedolizumab, ustekinumab, tofacitinib, fecal microbiota transplantation, leukocyte apheresis, and the Chinese herbal medicine Wuwei Kushen Enteric–coated capsules. Surgery-related risk assessments (5/30, 17%) primarily aimed to predict pouchitis, postoperative complications, and long-term surgical outcomes. Disease progression and relapse prediction (6/30, 20%) focused on risks of acute severe UC, relapse probabilities, and remission likelihoods (Multimedia Appendix 2).

Most studies (19/30, 63%) did not specify how missing data were handled. Among the remaining studies, the most common approach was direct deletion (6/30, 20%), whereas others used random forest (1/30, 3%), k-nearest neighbors (1/30, 3%), mean imputation (2/30, 7%), and multiple imputations (1/30, 3%). Data splitting into training and validation sets was the most common method, with a 7:3 split used in 6 studies. Predictor selection methods included regression techniques (13/30, 43%), such as logistic regression (6/30, 20%), LASSO regression (4/30, 13%), Cox regression (1/30, 3%), and elastic net regularization regression (1/30, 3%). Machine learning methods were used in 6 studies (6/30, 20%), comprising random forest (5/30, 17%) and CatBoost (1/30, 3%).

Common predictors across studies were age, C-reactive protein (CRP), sex, albumin, erythrocyte sedimentation rate, platelet count, hemoglobin, disease extent or severity, endoscopic findings, and Mayo scores. Logistic regression was the most frequently used modeling method (12/30, 40%), followed by neural networks (8/30, 27%) and random forest (3/30, 10%). Cross-validation was used in 17 studies (17/30, 57%), with 5-fold cross-validation being the most common. Bootstrap validation was performed in 9 studies (9/30, 30%). 12 studies (12/30, 40%) provided calibration curves, 3 (3/30, 10%) included decision curve analysis (DCA), and 12 (12/30, 40%) developed decision-support tools. Most studies adequately discussed their limitations, which included small sample sizes, single-center designs, retrospective biases, and lack of external validation (Multimedia Appendix 3).

### Quality Assessment

#### Overview

Table 1 presents a summary of the risk of bias and applicability for the included studies. Of the 30 studies, 29 were assessed as having a high risk of bias, whereas only 1 was rated as low risk.

**Table 1.** Risk of bias and applicability assessment: comprehensive evaluation of participants, predictors, and analytical methods.

Author, year	Risk of bias				Applicability			Overall	
	Participants	Predictors	Outcome	Analysis	Participants	Predictors	Outcome	Risk of bias	Applicability
Croft et al, 2024 [14]	+^a^	+	+	+	−^b^	+	+	+	−
Zhang et al, 2024 [15]	−	+	+	+	+	+	+	−	+
Iacucci et al, 2023 [16]	+	+	+	−	+	+	+	−	+
Chen et al, 2021 [17]	+	+	+	−	+	+	+	−	+
Morilla et al, 2019 [18]	−	+	+	−	−	+	+	−	−
Morilla et al, 2021 [19]	−	+	+	−	−	+	+	−	−
Takayama et al, 2015 [20]	−	+	+	?^c^	+	+	+	−	+
Bu et al, 2023 [21]	−	+	+	−	+	+	+	−	+
Li et al, 2022 [22]	−	+	+	?	+	+	+	−	+
Dai et al, 2024 [23]	−	+	+	?	+	+	+	−	+
Chen et al, 2023 [24]	−	+	+	−	+	+	+	−	+
Yu et al, 2022 [25]	−	+	+	?	−	+	+	−	−
Kang et al, 2022 [26]	+	+	+	−	−	+	+	−	−
Waljee et al, 2018 [27]	−	+	+	−	−	+	+	−	−
Miyoshi et al, 2021 [28]	−	+	+	?	−	+	+	−	−
Morikubo et al, 2024 [29]	−	+	+	−	−	+	+	−	−
Ghiassian et al, 2022 [30]	−	+	+	−	−	+	+	−	−
Sofo et al, 2020 [31]	−	+	?	−	−	−	+	−	−
Feng et al, 2021 [32]	−	+	+	−	−	+	+	−	−
Konikoff et al, 2024 [33]	−	+	+	−	−	+	+	−	−
Cesarini et al, 2017 [34]	−	+	+	+	+	+	+	−	+
Derakhshan Nazari et al, 2023 [35]	+	+	+	−	−	+	+	−	−
Kim et al, 2023 [36]	+	+	+	−	+	+	+	−	+
Lees et al, 2021 [37]	+	+	+	−	−	+	+	−	−
Ghoshal et al, 2020 [38]	−	+	?	?	−	+	+	−	−
Mizuno et al, 2022 [39]	−	+	+	−	−	+	+	−	−
Pang et al, 2023 [40]	−	+	+	−	+	+	+	−	+
Chen et al, 2022 [41]	+	+	+	−	−	+	+	−	−
Wang et al, 2023 [42]	−	+	+	−	−	+	+	−	−
Wang et al, 2023 [43]	−	+	+	−	+	+	+	−	+

a “+”: low risk of bias or low concern regarding applicability.

b “−”: high risk of bias or high concern regarding applicability.

c “?”: unclear risk of bias or unclear concern regarding applicability.

- Participants domain: 22 studies (22/30, 73%) were rated as high risk due to their retrospective cohort design.
- Predictors and outcomes domains: all studies adequately reported these aspects and were rated as having a low risk of bias.
- Analysis domain: 11 studies (11/30, 37%) had an events-per-variable ratio below 10 or a validation sample size smaller than 100. One study (1/30, 3%) did not report sample size estimation. Fourteen studies (14/30, 5%) did not assess model calibration or discrimination, and 4 (4/30, 13%) lacked sufficient information on either. Consequently, 21 studies (21/30, 70%) were rated as having a high risk of bias in this domain, and 6 studies (6/30, 20%) were rated as having unclear risk. In terms of applicability, 18 studies (18/30, 60%) were rated as having high concerns, whereas 12 (12/30, 40%) were rated as having low concerns. Participants domain: 18 studies (18/30, 60%) were judged to have high applicability concerns because of their focus on specific subgroups of patients with UC.
- Predictors domain: 1 study (1/30, 3%) had high applicability concerns due to unreported follow-up times.
- Outcomes domain: all studies had outcomes consistent with the systematic review question and were rated as having low applicability concerns.

#### Model Validation and Meta-Analysis

Among the included studies, 16 (16/30, 53%) conducted internal validation, 14 (14/30, 47%) performed external validation, and only 6 (6/30, 20%) provided both internal and external validation. 5 (5/30, 17%) studies provided 95% CIs for internal validation AUC, and 4 (4/30, 13%) reported 95% CIs for external validation AUC. Only 2 studies (2/30, 7%) fully reported both internal and external validation AUC values along with their 95% CIs.

We conducted a meta-analysis of AUC values and their 95% CIs, with subgroup analyses for internal and external validation. The overall pooled AUC was 0.84 (95% CI 0.77‐0.92). For internal validation, the pooled AUC was 0.83 (95% CI 0.70‐0.96), whereas for external validation, it was 0.87 (95% CI 0.78‐0.95). High heterogeneity was observed in internal validation results (Multimedia Appendix 4). Funnel plots and the Egger regression analysis indicated potential publication bias in internal validation results. Sensitivity analysis by sequentially removing individual studies revealed that excluding the study by Kim et al [36] significantly reduced heterogeneity. Detailed examination of the Kim et al [36] study revealed that its small sample size likely introduced random errors, leading to model overfitting and inflated performance estimates. After excluding this study, the pooled AUC for internal validation was recalculated as 0.78 (95% CI 0.74‐0.81; Multimedia Appendix 4a).

Owing to the limited reporting of sensitivity and specificity values along with their 95% CIs, we calculated the average sensitivity and specificity values for both internal and external validation. For internal validation, the average sensitivity and specificity were 0.814 and 0.761, respectively (8/30, 27% and 7/30, 23%, respectively). For external validation, the average sensitivity and specificity were 0.830 and 0.757, respectively (11/30, 37% and 9/30, 30%, respectively).

## Discussion

### Principal Findings

We systematically reviewed 30 studies on UC prognostic models. Most studies focused on predicting treatment outcomes, disease progression or relapse, and surgery-related risks, especially the effectiveness of biologic therapies such as TNF-α inhibitors. Logistic regression was the most commonly used method for predictor selection and model construction because of its simplicity, interpretability, and ability to provide insights into the relationships between predictors and outcomes [44]. Common predictors across most UC prognostic models included age, CRP, sex, albumin, erythrocyte sedimentation rate, platelet count, hemoglobin, disease extent/severity, endoscopic findings, and Mayo scores. Prior research has indicated a strong link between age and UC prognosis [45], whereas CRP has been recognized as an effective, noninvasive biomarker for evaluating treatment response in patients with UC [46]. Most studies did not clearly report how missing data were handled, and few developed decision support tools (eg, calculators or nomograms). Small sample size was a common limitation across the majority of studies.

The meta-analysis reported pooled AUCs of 0.84 (95% CI 0.77‐0.92) for overall validation, 0.83 (95% CI 0.70‐0.96) for internal validation, and 0.87 (95% CI 0.78‐0.95) for external validation. A notable and somewhat unexpected finding was that models evaluated through external validation demonstrated a higher pooled AUC than those assessed internally. This contradicts conventional expectations, as external validation typically yields lower predictive performance due to increased heterogeneity and real-world variability. Several factors may account for this discrepancy. First, further analysis of the 4 studies (4/30, 13%) in the external validation subgroup revealed several shared characteristics. Some used advanced modeling techniques, such as convolutional neural networks and artificial neural networks, which may have contributed to improved predictive performance. Additionally, the outcomes predicted by these models, such as drug sustainability or short-term treatment response, were inherently more predictable, which may have facilitated higher accuracy. Second, publication bias may have contributed to the observed results. Studies reporting poor performance in external validation may have remained unpublished or underreported, thereby inflating the pooled estimates. Third, the number of studies reporting external validation AUCs was relatively small (4/30, 13%), which may have introduced statistical uncertainty and led to overestimation of model performance. Moreover, according to the PROBAST risk of bias assessment, all 4 studies (4/30, 13%) were rated as having a high overall risk of bias. Therefore, although the pooled AUC from external validation appears high, this result should be interpreted with caution due to the limited number of studies, the high risk of bias, and the potential for selective reporting. Regarding sensitivity and specificity, the mean values for internal validation were 0.814 and 0.761, respectively, and for external validation, 0.830 and 0.757. It is important to note that these values were derived using simple unweighted averages due to the inconsistent reporting of 95% CIs across studies. Consequently, this approach does not account for variations in sample size or the precision of estimates, which may introduce bias. However, significant heterogeneity was observed across the included studies, particularly for internal validation. Sensitivity analysis indicated that studies with small sample sizes may have overestimated model performance due to overfitting. This finding underscores the importance of prioritizing high-quality training data and conducting appropriate sample size estimations during model development [47]. Some studies reported calibration curves and DCA, indicating the potential utility of these models in specific clinical scenarios. However, only a minority of studies comprehensively reported performance metrics (eg, 95% CIs for AUC, sensitivity, and specificity), limiting the comparability and robustness of the results.

The PROBAST tool–based quality assessment showed that the majority of studies carried a high risk of bias, especially in aspects concerning participant selection and model analysis. Many studies were limited to specific UC subgroups (eg, moderate-to-severe cases or patients receiving specific treatments), reducing the external applicability of the results. Additionally, some studies did not report sample size calculations or follow-up durations, further restricting the generalizability of the models. This limitation may lead to insufficient evaluation of model performance and reduce the credibility of the results. The lack of standardized approaches for predictor selection and the absence of explicit methods for handling missing data were notable limitations. The frequent use of direct deletion to address missing data may have introduced sample bias. Although 14 studies (14/30, 47%) conducted external validation, most validation datasets were small and lacked multicenter data. Only 2 studies (2/30, 7%) conducted both internal and external validation, raising concerns about the reliability and generalizability of current UC prognostic models in external settings. One study, which focused on a specific population, was identified as having a high risk of limited applicability [14]. However, it demonstrated a low risk of bias, underscoring its potential for future validation in targeted populations.

### Comparison to Prior Work

Artificial intelligence has attracted growing attention in the diagnosis and management of UC. Previous research has primarily concentrated on diagnostic models. For example, Jahagirdar et al [48] conducted a meta-analysis evaluating convolutional neural network–based algorithms for predicting endoscopic severity, whereas Puga-Tejada et al [49] examined artificial intelligence performance in detecting histological remission. However, despite their promise for real-time assessment, these studies primarily address current activity and overlook long-term outcomes.

In contrast, our study is the first to systematically review and meta-analyze prognostic prediction models for UC, focusing on outcomes, such as treatment response, relapse, disease progression, and the need for surgery. We used the PROBAST tool to evaluate model quality rigorously and synthesized key performance measures, including the AUC, sensitivity, and specificity. Furthermore, we assessed model calibration, decision-support tools, and the completeness of reporting. Unlike previous reviews, our study highlights the predictive utility and clinical relevance of prognostic models, providing valuable insights for advancing precision medicine in UC.

### Future Directions

Future studies should prioritize the complete and transparent reporting of performance metrics, such as AUC, sensitivity, specificity, and their 95% CIs, to enable more reliable and meaningful evaluations of model utility. Additionally, researchers should incorporate appropriate sample size calculations and clearly report follow-up durations to improve methodological rigor and ensure reproducibility [50]. Addressing these limitations will enhance the overall quality, transparency, and clinical applicability of predictive models for UC.

In light of the growing significance of prediction models in UC prognosis, we recommend that future research adhere to the Transparent Reporting of a Multivariable Prediction Model for Individual Prognosis or Diagnosis reporting guidelines and adopt the 9-step framework for developing and validating clinical prediction models. These frameworks emphasize best practices, including clearly defining the target population and intended end users, selecting high-quality data sources, properly handling missing data, exploring alternative modeling approaches, and rigorously assessing model performance through both internal and external validation [51,52]. For instance, when defining the target population (step 1 of the 9-step framework), researchers should clearly distinguish between patients with newly diagnosed UC and those with long-standing disease, as their clinical trajectories, risk profiles, and treatment responses may differ significantly. During model development, step 4 (handling missing data) is particularly relevant in UC research, where laboratory and endoscopic variables are frequently incomplete. Therefore, appropriate statistical approaches, such as multiple imputation, should be used in place of listwise deletion, which can introduce bias and reduce statistical power. For step 8 (external validation), it is crucial to evaluate model performance using datasets from distinct geographic regions or health care systems. For example, a model developed in a tertiary care center in East Asia could be externally validated using a population-based registry from Europe or North America. This approach helps assess the model’s generalizability across diverse UC populations. Furthermore, in alignment with Transparent Reporting of a Multivariable Prediction Model for Individual Prognosis or Diagnosis recommendations, future studies should ensure transparent reporting of all aspects of model development, including predictor selection, handling of missing data, and strategies to address overfitting. Reporting should also include calibration plots, discrimination metrics (eg, AUC or C-index), and their 95% CIs to support comprehensive and interpretable performance evaluation.

### Limitations

This study has several limitations. First, substantial heterogeneity existed among the included studies in terms of study design, population characteristics, modeling approaches, and outcome definitions, which may have affected comparability and introduced variability into the pooled estimates. Second, external validation was limited, with most models relying on small or single-center datasets, thereby restricting their generalizability. Third, many studies did not consistently report key performance metrics, such as 95% CIs for AUC, sensitivity, and specificity, limiting the ability to critically evaluate and compare model performance. Fourth, missing data were often poorly addressed, with many studies using complete-case analysis or listwise deletion, which increases the risk of bias and reduces statistical power. Finally, we only included studies published in English or Chinese and searched 8 major databases, which may have introduced language bias and led to the omission of relevant studies from other languages, sources, or the grey literature.

### Conclusions

This study identified 30 prognostic prediction models for UC, encompassing 7452 patients, with most studies conducted in China and Japan. The majority of studies were retrospective and multicenter, primarily aimed at predicting therapeutic responses, particularly to TNF-α inhibitors, such as infliximab and adalimumab, as well as estimating the risks of surgery, disease progression, or relapse. Logistic regression was the most frequently used method for predictor selection and model development, with commonly used predictors, including age, CRP, albumin, hemoglobin, disease extent, and Mayo scores. The meta-analysis revealed a pooled AUC of 0.84 (95% CI 0.77-0.92). However, notable limitations were identified, including a high risk of bias in most studies, insufficient external validation, and restricted generalization due to small sample sizes and subgroup-specific designs. These findings underscore the need for future research to prioritize robust model development, rigorous external validation, and enhanced generalization to support broader clinical application. Ultimately, well-developed and validated prognostic models hold the potential to guide personalized treatment strategies, enhance clinical decision-making, and improve outcomes for patients with UC in real-world settings.

## Supplementary material

10.2196/71944 Multimedia Appendix 1 Detailed search strategy for all included databases (PubMed, Embase, Cochrane Library, Web of Science, SinoMed, CNKI, Wanfang, VIP).

10.2196/71944 Multimedia Appendix 2 Comprehensive summary of study characteristics: study design, objectives, and subgroup descriptions of patients with ulcerative colitis.

10.2196/71944 Multimedia Appendix 3 Detailed summary of predictive model characteristics: variable selection methods, model construction techniques, and validation results.

10.2196/71944 Multimedia Appendix 4 Meta-analysis results on internal validation of area under the curve (AUC) values.

10.2196/71944 Checklist 1 PRISMA 2020 checklist.

## Abbreviations

AUC: area under the curve
C-index: concordance index
CRP: C-reactive protein
DCA: decision curve analysis
PICOS: population, intervention, comparator, outcome, and study design
PRISMA: Preferred Reporting Items for Systematic Reviews and Meta-Analyses
PROBAST: Prediction Model Risk of Bias Assessment Tool
TNF-α: tumor necrosis factor-alpha
UC: ulcerative colitis
