# Supplementary material for: Prognostic Prediction Models for Ulcerative Colitis: Systematic Review and Meta-Analysis
Source: J Med Internet Res. 2025 Dec 22;27:e71944. doi: 10.2196/71944 (PMC12721486; doi:10.2196/71944)
Supplement: Multimedia Appendix 1 [file jmir-v27-e71944-s001.docx]

**Table S1.** Search strategy for PubMed

| # | Searches |
| --- | --- |
| 1 | "Colitis, Ulcerative"[Mesh] |
| 2 | ((((Colitis, Ulcerative[Title/Abstract]) OR (Colitis Gravis[Title/Abstract])) OR (Idiopathic Proctocolitis[Title/Abstract])) OR (Inflammatory Bowel Disease, Ulcerative Colitis Type[Title/Abstract])) OR (Ulcerative Colitis[Title/Abstract]) |
| 3 | (((((Colitis, Ulcerative[Title/Abstract]) OR (Colitis Gravis[Title/Abstract])) OR (Idiopathic Proctocolitis[Title/Abstract])) OR (Inflammatory Bowel Disease, Ulcerative Colitis Type[Title/Abstract])) OR (Ulcerative Colitis[Title/Abstract])) OR ("Colitis, Ulcerative"[Mesh]) |
| 4 | "Nomograms"[Mesh] |
| 5 | (((((((((Nomograms[Title/Abstract]) OR (Nomogram[Title/Abstract])) OR (Partin Tables[Title/Abstract])) OR (Partin Table[Title/Abstract])) OR (Table, Partin[Title/Abstract])) OR (Tables, Partin[Title/Abstract])) OR (Partin Nomograms[Title/Abstract])) OR (Nomogram, Partin[Title/Abstract])) OR (Nomograms, Partin[Title/Abstract])) OR (Partin Nomogram[Title/Abstract]) |
| 6 | "Machine Learning"[Mesh] |
| 7 | (((Machine Learning[Title/Abstract]) OR (Learning, Machine[Title/Abstract])) OR (Transfer Learning[Title/Abstract])) OR (Learning, Transfer[Title/Abstract]) |
|  | "Deep Learning"[Mesh] |
|  | "Neural Networks, Computer"[Mesh] |
|  | (((((Computer Neural Network[Title/Abstract]) OR (Computer Neural Networks[Title/Abstract])) OR (Network, Computer Neural[Title/Abstract])) OR (Networks, Computer Neural[Title/Abstract])) OR (Neural Network, Computer[Title/Abstract])) OR ((((Models, Neural Network[Title/Abstract]) OR (Model, Neural Network[Title/Abstract])) OR (Network Model, Neural[Title/Abstract])) OR (Network Models, Neural[Title/Abstract])) OR (Neural Network Model[Title/Abstract])) OR ((((Neural Network Models[Title/Abstract]) OR (Connectionist Models[Title/Abstract])) OR (Connectionist Model[Title/Abstract])) OR (Model, Connectionist[Title/Abstract])) OR (Models, Connectionist[Title/Abstract])) OR ((((Perceptrons[Title/Abstract]) OR (Perceptron[Title/Abstract])) OR (Computational Neural Networks[Title/Abstract])) OR (Computational Neural Network[Title/Abstract])) OR ((((Network, Computational Neural[Title/Abstract]) OR (Networks, Computational Neural[Title/Abstract])) OR (Neural Network, Computational [Title/Abstract])) OR (Neural Networks, Computational[Title/Abstract])) OR ((((Neural Networks [Title/Abstract]) OR (Network, Neural [Title/Abstract])) OR (Networks, Neural [Title/Abstract])) OR (Neural Network [Title/Abstract])) OR ((((Deep Learning[Title/Abstract]) OR (Neural Networks[Title/Abstract])) OR (Convolutional Neural Networks[Title/Abstract])) OR (Recurrent Neural Networks[Title/Abstract])) OR ((((Long Short-Term Memory[Title/Abstract]) OR (Deep Neural Networks[Title/Abstract])) OR ("Deep Learning"[Mesh])) OR ("Neural Networks, Computer"[Mesh])) |
| 8 | ((predict model[Title/Abstract]) OR (Forecast predict [Title/Abstract])) |
| 9 | ((((((predict model[Title/Abstract]) OR (Forecast model[Title/Abstract])) OR ((((Machine Learning[Title/Abstract]) OR (Learning, Machine[Title/Abstract])) OR (Transfer Learning[Title/Abstract])) OR (Learning, Transfer[Title/Abstract]))) OR ("Machine Learning"[Mesh])) OR ((((((((((Nomograms[Title/Abstract]) OR (Nomogram[Title/Abstract])) OR (Partin Tables[Title/Abstract])) OR (Partin Table[Title/Abstract])) OR (Table, Partin[Title/Abstract])) OR (Tables, Partin[Title/Abstract])) OR (Partin Nomograms[Title/Abstract])) OR (Nomogram, Partin[Title/Abstract])) OR (Nomograms, Partin[Title/Abstract])) OR (Partin Nomogram[Title/Abstract]))) OR ("Nomograms"[Mesh]) |
| 13 | (((((((predict model[Title/Abstract]) OR (Forecast model[Title/Abstract])) OR ((((Machine Learning[Title/Abstract]) OR (Learning, Machine[Title/Abstract])) OR (Transfer Learning[Title/Abstract])) OR (Learning, Transfer[Title/Abstract]))) OR ("Machine Learning"[Mesh])) OR ((((((((((Nomograms[Title/Abstract]) OR (Nomogram[Title/Abstract])) OR (Partin Tables[Title/Abstract])) OR (Partin Table[Title/Abstract])) OR (Table, Partin[Title/Abstract])) OR (Tables, Partin[Title/Abstract])) OR (Partin Nomograms[Title/Abstract])) OR (Nomogram, Partin[Title/Abstract])) OR (Nomograms, Partin[Title/Abstract])) OR (Partin Nomogram[Title/Abstract]))) OR ("Nomograms"[Mesh])) OR ((((((Computer Neural Network[Title/Abstract]) OR (Computer Neural Networks[Title/Abstract])) OR (Network, Computer Neural[Title/Abstract])) OR (Networks, Computer Neural[Title/Abstract])) OR (Neural Network, Computer[Title/Abstract])) OR ((((Models, Neural Network[Title/Abstract]) OR (Model, Neural Network[Title/Abstract])) OR (Network Model, Neural[Title/Abstract])) OR (Network Models, Neural[Title/Abstract])) OR (Neural Network Model[Title/Abstract])) OR ((((Neural Network Models[Title/Abstract]) OR (Connectionist Models[Title/Abstract])) OR (Connectionist Model[Title/Abstract])) OR (Model, Connectionist[Title/Abstract])) OR (Models, Connectionist[Title/Abstract])) OR ((((Perceptrons[Title/Abstract]) OR (Perceptron[Title/Abstract])) OR (Computational Neural Networks[Title/Abstract])) OR (Computational Neural Network[Title/Abstract])) OR ((((Network, Computational Neural[Title/Abstract]) OR (Networks, Computational Neural[Title/Abstract])) OR (Neural Network, Computational[Title/Abstract])) OR (Neural Networks, Computational[Title/Abstract])) OR ((((Neural Networks [Title/Abstract]) OR (Network, Neural [Title/Abstract])) OR (Networks, Neural [Title/Abstract])) OR (Neural Network [Title/Abstract])) OR ((((Deep Learning[Title/Abstract]) OR (Neural Networks[Title/Abstract])) OR (Convolutional Neural Networks[Title/Abstract])) OR (Recurrent Neural Networks[Title/Abstract])) OR ((((Long Short-Term Memory[Title/Abstract]) OR (Deep Neural Networks[Title/Abstract])) OR ("Deep Learning"[Mesh])) OR ("Neural Networks, Computer"[Mesh])) AND ((((((Colitis, Ulcerative[Title/Abstract]) OR (Colitis Gravis[Title/Abstract])) OR (Idiopathic Proctocolitis[Title/Abstract])) OR (Inflammatory Bowel Disease, Ulcerative Colitis Type[Title/Abstract])) OR (Ulcerative Colitis[Title/Abstract])) OR ("Colitis, Ulcerative"[Mesh])) |

**Table S2.** Search strategy for the Cochrane Central Register of Controlled Trials (CENTRAL)

| # | Searches |
| --- | --- |
| 1 | MeSH descriptor: [Colitis, Ulcerative] explode all trees |
| 2 | (Colitis, Ulcerative):ti,ab,kw OR (Idiopathic Proctocolitis):ti,ab,kw OR (Inflammatory Bowel Disease):ti,ab,kw OR (Ulcerative Colitis Type):ti,ab,kw OR (Ulcerative Colitis):ti,ab,kw |
| 3 | (Colitis Gravis):ti,ab,kw |
| 4 | #1 OR #2 OR #3 |
| 5 | MeSH descriptor: [Nomograms] explode all trees |
| 6 | (Nomograms):ti,ab,kw OR (Nomograms, Partin):ti,ab,kw OR (Table, Partin):ti,ab,kw OR (Partin Nomograms):ti,ab,kw OR (Nomogram, Partin):ti,ab,kw |
| 7 | (Partin Tables):ti,ab,kw OR (Partin Table):ti,ab,kw OR (Tables, Partin):ti,ab,kw OR (Partin Nomogram):ti,ab,kw OR (Nomogram):ti,ab,kw |
| 8 | MeSH descriptor: [Machine Learning] explode all trees |
| 9 | (Machine Learning):ti,ab,kw OR (Learning, Machine):ti,ab,kw OR (Transfer Learning):ti,ab,kw OR (Learning, Transfer):ti,ab,kw |
| 10 | (predict model):ti,ab,kw OR (Forecast model):ti,ab,kw |
| 11 | #5 OR #6 OR #7 OR #8 OR #9 OR #10 |
| 12 | MeSH descriptor: [Deep Learning] explode all trees |
| 13 | (Deep Learning):ti,ab,kw OR (Learning, Deep):ti,ab,kw OR (Learning, Hierarchical):ti,ab,kw OR (Hierarchical Learning):ti,ab,kw |
| 14 | (Neural Networks):ti,ab,kw OR (Convolutional Neural Networks):ti,ab,kw OR (Recurrent Neural Networks):ti,ab,kw OR (Long Short-Term Memory):ti,ab,kw OR (Deep Neural Networks):ti,ab,kw |
| 15 | #12 OR #13 OR #14 |
| 16 | #11 OR #15 |
| 17 | #4 AND #16 |

**Table S3.** Search strategy for Embase

| # | Searches |
| --- | --- |
| 1 | 'ulcerative colitis'/exp |
| 2 | 'chronic ulcerative colitis':ab,ti OR 'colitis  ulcerativa':ab,ti OR 'colitis ulcerosa':ab,ti OR  'colitis ulcerosa chronica':ab,ti OR 'colitis,  mucosal':ab,ti OR 'colitis, ulcerative':ab,ti OR  'colitis, ulcerous':ab,ti OR 'colon, chronic  ulceration':ab,ti OR 'histiocytic ulcerative  colitis':ab,ti OR 'mucosal colitis':ab,ti OR  'ulcerative colorectitis':ab,ti OR 'ulcerative  procto colitis':ab,ti OR 'ulcerative  proctocolitis':ab,ti OR 'ulcerous colitis':ab,ti  OR 'ulcerative colitis':ab,ti |
| 3 | 'nomogram'/exp |
| 4 | 'nomograms':ab,ti OR 'nomograph':ab,ti OR  'nomogram':ab,ti |
| 5 | 'machine learning'/exp |
| 6 | 'learning machine':ab,ti OR 'learning machines':ab,ti OR 'machine learning':ab,ti |
| 7 | 'predict model':ab,ti OR 'forecast model':ab,ti |
| 8 | 'deep learning'/exp |
| 9 | 'deep machine learning':ab,ti OR 'deep ml':ab,ti OR 'hierarchical learning':ab,ti OR 'deep learning':ab,ti OR 'neural networks':ab,ti OR 'convolutional neural networks':ab,ti OR 'long short-term memory':ab,ti OR 'deep neural networks':ab,ti |
| 10 | #1 OR #2 |
| 11 | #3 OR #4 OR #5 OR #6 OR #7 OR #8 OR #9 |
| 12 | #10 AND #11 |

**Table S4.** Search strategy for Web of Science: Science Citation Index Expanded

| # | Searches |
| --- | --- |
| 1 | TS=("Colitis, Ulcerative" OR "Colitis Gravis" OR "Idiopathic Proctocolitis" OR "Inflammatory Bowel Disease, Ulcerative Colitis Type" OR "Ulcerative Colitis") |
| 2 | TS=("predict model" OR "Forecast model" OR "Machine Learning" OR "Learning, Machine" OR "Transfer Learning" OR "Learning, Transfer" OR "Nomograms" OR "Nomogram" OR "Partin Tables" OR "Partin Table" OR "Table, Partin" OR "Tables, Partin" OR "Partin Nomograms" OR "Nomogram, Partin" OR "Nomograms, Partin" OR "Partin Nomogram" OR "Deep Learning" OR "Neural Networks" OR "Convolutional Neural Networks" OR "Recurrent Neural Networks" OR "Long Short-Term Memory" OR "Deep Neural Networks" ) |
| 4 | #1 AND #2 |

**Table S5.** Search strategy for CNKI

| **#** | Searches |
| --- | --- |
| 1 | TKA=('溃疡性结肠炎' + '结肠炎,溃疡性' + '直肠结肠炎' + '溃疡性直肠结肠炎' + '溃疡性直结肠炎' + '直结肠炎' + '直结肠炎,溃疡性') AND TKA=('预测模型' + '机器学习' + '列线图' + '诺莫图'+'Nomogram'+'深度学习'+'神经网络'+'卷积神经网络'+'递归神经网络'+'长短期记忆'+'深度神经网络'+'多模态') |

**Table S6.** Search strategy for WangFang

| **#** | Searches |
| --- | --- |
| 1 | (题名或关键词:(溃疡性结肠炎 or 结肠炎, 溃疡性 or 直肠结肠炎 or 溃疡性直肠结肠炎 or 溃疡性直结肠炎 or 直结肠炎 or 直结肠炎,溃疡性) or 摘要:(溃疡性结肠炎 or 结肠炎, 溃疡性 or 直肠结肠炎 or 溃疡性直肠结肠炎 or 溃疡性直结肠炎 or 直结肠炎 or 直结肠炎,溃疡性)) and (题名或关键词:(预测模型 or 机器学习 or 列线图 or 诺莫图 or Nomogram or 深度学习 or 神经网络 or 卷积神经网络 or 递归神经网络 or 长短期记忆 or深度神经网络 or 多模态) or 摘要:(预测模型 or 机器学习 or 列线图 or 诺莫图 or Nomogram or 深度学习 or 神经网络 or 卷积神经网络 or 递归神经网络 or 长短期记忆 or深度神经网络 or 多模态) ) |

**Table S7.** Search strategy for VIP

| **#** | Searches |
| --- | --- |
| 1 | M=(溃疡性结肠炎 or 结肠炎,溃疡性 or 直肠结肠炎 or 溃疡性直肠结肠炎 or 溃疡性直结肠炎 or 直结肠炎 or 直结肠炎,溃疡性) and M=(预测模型 or 机器学习 or 列线图 or 诺莫图 or Nomogram or 深度学习 or 神经网络 or 卷积神经网络 or 递归神经网络 or 长短期记忆 or深度神经网络 or 多模态) |

**Table S8.** Search strategy for Sinomed

| **#** | Searches |
| --- | --- |
| 1 | "结肠炎,溃疡性"[不加权:扩展] |
| 2 | "溃疡性结肠炎"[常用字段:智能] OR "结肠炎,溃疡性"[常用字段:智能] OR "直肠结肠炎"[常用字段:智能] OR "溃疡性直肠结肠炎"[常用字段:智能] OR "直结肠炎"[常用字段:智能] OR "直结肠炎,溃疡性"[常用字段:智能] |
| 3 | ("列线图"[不加权:扩展]) OR "预测"[不加权:扩展] |
| 4 | "预测模型"[常用字段:智能] OR "列线图"[常用字段:智能] OR "诺莫图"[常用字段:智能] |
| 5 | "复发"[不加权:扩展] |
| 6 | "复发"[常用字段:智能] OR "再发"[常用字段:智能] OR "再作"[常用字段:智能] OR "再发作"[常用字段:智能] |
| 7 | "鼠"[常用字段:智能] OR "研究进展"[常用字段:智能] OR "Meta"[常用字段:智能] OR "指南"[常用字段:智能] OR "孟德尔"[常用字段:智能] OR "经验"[常用字段:智能] OR "综述"[常用字段:智能] OR "随机对照"[常用字段:智能] |
| 8 | (#2) OR (#1) |
| 9 | (#4) OR (#3) |
| 10 | (#6) OR (#5) |
| 11 | (#10) AND (#9) AND (#8) |
| 12 | (#11) NOT (#7) |
