# Supplementary material for: Prognostic Prediction Models for Ulcerative Colitis: Systematic Review and Meta-Analysis
Source: J Med Internet Res. 2025 Dec 22;27:e71944. doi: 10.2196/71944 (PMC12721486; doi:10.2196/71944)
Supplement: Multimedia Appendix 2 [file jmir-v27-e71944-s002.docx]

| **Author**  Multimedia Appendix 2. Comprehensive summary of study characteristics: Study design, objectives, and subgroup descriptions of ulcerative colitis patients. | **Year** | **Population source** | **Single-Center or Multi-Center study** | **Study design** | **Specific subgroup of ulcerative colitis patients** | **Total sample size** | **Study objective** |
| --- | --- | --- | --- | --- | --- | --- | --- |
| Anthony Croft [14] | 2024 | Australia | Single-center | Prospective | Mainly severe ulcerative colitis (UC)^a^ | 682 | Personalized algorithm to predict the risk of intravenous corticosteroid treatment failure in severe UC patients |
| Sheng Zhang [15] | 2024 | China | Single-center | Retrospective | Mainly moderate-to-severe UC | 366 | Predicting clinical response one month after fecal microbiota transplantation in UC patients |
| Marietta Iacucci [16] | 2022 | Multiple countries | Multi-center study | Prospective | Not explicitly defined | 283 | Artificial intelligence based virtual staining endoscopy system to detect endoscopic/ histological activity in UC and predict 12-month outcomes (eg, surgery, hospitalization, treatment adjustment) |
| Xuanfu Chen [17] | 2021 | China | Multi-center study | Prospective + Retrospective | Not explicitly defined | 97 | Artificial neural network analysis combined with immune-related genes to predict primary non-response to infliximab in UC patients |
| Ian Morilla [18] | 2019 | France | Multi-center study | Retrospective | Severe UC patients | 76 | Combining microRNA and clinical parameters to predict the efficacy of first- and second-line treatments (intravenous steroids, cyclosporine, or infliximab) in acute severe UC (ASUC) ^b^ patients |
| Ian Morilla [19] | 2021 | France | Multi-center study | Retrospective | Not explicitly defined | 57 | Combining intraoperative microRNA and clinical factors to predict the occurrence of pouchitis in UC patients undergoing ileal pouch surgery |
| Tetsuro Takayama [20] | 2015 | Japan | Multi-center study | Retrospective | Moderate-to-severe UC patients | 90 | Development of an artificial neural network (ANN)^c^ model to predict long-term outcomes (eg, surgical risk) after leukocytapheresis in UC patients. |
| Zhijun Bu [21] | 2023 | China | Multi-center study | Retrospective | Not explicitly defined | 274 | Predicting the probability of disease remission in UC patients based on treatment data with five-flavor sophora enteric-coated capsule (FSEC)^d^ |
| Na Li [22] | 2022 | China | Single-center | Retrospective | Not explicitly defined | 313 | Predicting whether UC patients have an indolent disease course |
| Cong Dai [23] | 2024 | China | Single-center | Retrospective | Not explicitly defined | 284 | To predict clinical non-response to 5-aminosalicylic acid (5-ASA)^e^ treatment in patients with UC |
| Jie Chen [24] | 2023 | China | Multi-center study | Retrospective | Not explicitly defined | 798 | Developing a risk model for predicting the progression of UC to ASUC within 1 year |
| Si Yu [25] | 2022 | China | Multi-center study | Retrospective | Severe UC patients | 194 | Predicting corticosteroid resistance in ASUC patients using clinical, laboratory, and endoscopic parameters |
| Gi-Ung Kang [26] | 2022 | South Korea | Single-center study | Prospective | Moderate-to-severe UC patients | 10 | Predicting clinical success of fecal microbiota transplantation based on gut microbiota composition in UC patients |
| Akbar K. Waljee [27] | 2018 | United States | Multi-center study | Retrospective | Moderate-to-severe UC patients | 491 | Predicting steroid-free endoscopic remission at 52 weeks in UC patients treated with vedolizumab |
| Jun Miyoshi [28] | 2021 | Japan | Multi-center study | Retrospective | Moderate-to-severe UC patients | 69 | Predicting the effectiveness of vedolizumab treatment at 22 weeks based on baseline clinical data in UC patients |
| Hiromu Morikubo [29] | 2024 | Japan | Multi-center study | Retrospective | Moderate-to-severe UC patients | 71 | Predicting the likelihood of steroid-free clinical remission at 22 weeks in UC patients treated with ustekinumab |
| Susan D. Ghiassian [30] | 2022 | Multiple countries | Multi-center study | Retrospective | Moderate-to-severe UC patients | 46 | Network-based molecular characterization for predicting non-response to tumor necrosis factor-alpha (TNF-α)^f^ inhibitors, such as infliximab, in patients with UC |
| Sofo [31] | 2020 | Italy | Single-center | Retrospective | Severe UC patients | 32 | Combining preoperative data with machine learning to predict postoperative complications in UC patients |
| Jing Feng [32] | 2021 | Multiple countries | Multi-center study | Retrospective | Moderate-to-severe UC patients | 74 | Gene-based prediction of primary non-response to infliximab in UC patients |
| Tom Konikoff [33] | 2024 | Israel | Multi-center study | Retrospective | Moderate-to-severe UC patients | 313 | Predicting drug sustainability in moderate-to-severe UC patients treated with infliximab and vedolizumab |
| Monica Cesarini [34] | 2017 | Multiple countries | Multi-center study | Retrospective | Not explicitly defined | 505 | Predicting the risk of developing ASUC within 3 y in UC patients |
| Mohammad Hossein Derakhshan Nazari [35] | 2023 | Multiple countries | Multi-center study | Prospective+ Retrospective | Moderate-to-severe UC patients | 79 | Developing a prediction tool based on multi-mRNA biomarkers and integrated machine learning models to predict response to anti-TNF therapy in UC patients |
| Seok-Young Kim [36] | 2024 | South Korea | Multi-center study | Prospective | Not explicitly defined | 62 | To develop a predictive model for clinical remission in patients with UC treated with adalimumab using Fourier-transform infrared spectroscopy and machine learning. |
| Charlie W. Lees [37] | 2021 | Multiple countries | Multi-center study | Prospective | Moderate-to-severe UC patients | 841 | Early treatment response to predict outcomes in UC patients treated with tofacitinib |
| Uday C. Ghoshal [38] | 2020 | India | Single-center | Retrospective | Severe UC patients | 263 | Predicting response to initial drug therapy in ASUC patients |
| Mizuno [39] | 2022 | Japan | Single-center | Retrospective | Not explicitly defined | 43 | Deep learning model to predict the risk of pouchitis after ileal pouch-anal anastomosis in UC patients |
| Wenwen Pang [40] | 2023 | China | Single-center | Retrospective | Not explicitly defined | 292 | Predicting UC relapse based on serum markers |
| Jingjing Chen [41] | 2022 | Multiple countries | Multi-center study | Prospective | Moderate-to-severe UC patients | 429 | Predicting clinical and health-related quality of life outcomes in moderate-to-severe UC patients treated with vedolizumab using supervised machine learning models |
| Zhongyuan Wang [42] | 2023 | China | Single-center | Retrospective | Severe UC patients | 142 | Predicting the risk of pouchitis after ileal pouch-anal anastomosis in UC patients |
| Xuehui Wang [43] | 2023 | China | Multi-center study | Retrospective | Not explicitly defined | 370 | Developing an efficacy prediction model for FSEC treatment in active UC patients |

^a^UC=ulcerative colitis

^b^ASUC=acute severe ulcerative colitis

^c^ANN=artificial neural network

^d^FSEC=five-flavor sophora enteric-coated capsule

^e^5-ASA=5-aminosalicylic acid

^f^TNF-α=tumor necrosis factor-alpha
