# Supplementary material for: Prognostic Prediction Models for Ulcerative Colitis: Systematic Review and Meta-Analysis
Source: J Med Internet Res. 2025 Dec 22;27:e71944. doi: 10.2196/71944 (PMC12721486; doi:10.2196/71944)
Supplement: Multimedia Appendix 3 [file jmir-v27-e71944-s003.docx]

| **Author**  Multimedia Appendix 3. Detailed summary of predictive model characteristics: Variable selection methods, model construction techniques, and validation results. | **Year** | **Missing Data Handling Method** | **Random Splitting Method** | **Variable Selection Method** | **Predictive Factors** | **Final Model Construction Method** | **Cross-Validation or Bootstrap Validation** | **AUC/C-index** | **Sensitivity/Specificity** | **Calibration Curve** | **Decision Curve Analysis** | **Presentation of Predictive Model** | **Discussion of Study Limitations** |
| --- | --- | --- | --- | --- | --- | --- | --- | --- | --- | --- | --- | --- | --- |
| Anthony Croft [14] | 2024 | Random forest imputation | Not reported | Akaike information criterion (AIC)^a^+ Elastic Net Regression | Prior use of oral corticosteroids, ≥6 bowel movements in 24 hours, albumin level, C-reactive protein (CRP)^b^ ≥12 mg/mL, log10- transformed CRP, Mayo endoscopic score, and disease extent | Logistic regression | 10-fold cross-validation (100 times) | Area under the curve (AUC^c^): 0.78 (95% confidence interval [CI]^d^: 0.74–0.82, internal validation); AUC: 0.78, 95%CI:0.71-0.85,external validation | Sensitivity/Specificity: 0.765; 0.663 (external validation) | Proximal calibration curve; good consistency between predicted probabilities and actual treatment failure rates | Not reported | Developed to predict intravenous corticosteroid failure in hospitalized ulcerative colitis (UC)^e^ patients; an online calculator is available at: <https://www.severcoli-tis.com> | Limitations include the single-center study design, small sample size, missing data for some variables, and validation of a follow-up on biologic therapy. |
| Sheng Zhang [15] | 2024 | k-nearest neighbors (KNN)^f^ imputation | 80-20 random split (training:testing) | Logistic regression, random forest, and collinearity analyses | Age, bowel movement frequency, Mayo score, platelet- large cell ratio, platelet distribution width, positive platelet count, bicarbonate ion, gamma-glutamyl transferase, indirect bilirubin, cholinesterase, creatine kinase, thrombin time, international normalized ratio, antithrombin III | Voting ensemble method | 5-fold cross-validation | AUC: 0.769 (internal validation), 0.614 (external validation) | Not reported | Not reported | Not reported | Developed to predict theefficacy ofwashedmicrobiotatransplantation in UC patients; an onlinecalculator is available at https://wmtpredict.strea mlit.app | Limitationsinclude thesingle-centerstudy design,limitedinterpretability of the machinelearningapproach, andmissing data for certain variables |
| Marietta Iacucci [16] | 2022 | Not reported | Not reported | Expert-labeled annotations used to define training labels | Endoscopic remission was defined as an UC Endoscopic Index of Severity (UCEIS)^h^ ≤1 or a Paddington International virtual ChromoendoSco py ScOre ≤3 | Convolutional neural network | Bootstrap validation (repeated resampling) | AUC: 0.94 (95% CI: 0.91–0.97; external validation) | Sensitivity: 0.79, Specificity: 0.95 (external validation) | Not reported | Not reported | Not reported | Limitations include variability in endoscopic video quality, subjective pathological evaluation, limited generalizability, and lack of real-world validation. |
| Xuanfu Chen [17] | 2021 | Not reported | Not reported | Differentially expressed gene analysis, robust rank aggregation, and immunohistochemistry | RNA-level features: CDX2, CHF2, and HSD11B2 expression; Protein-level features: RANK and VDR expression, | Artificial neural network | Reordering validation (500 iterations) | AUC: 0.850 (internal validation), 0.759 (external validation) | Not reported | Not reported | Not reported | Not reported | Limitations include small sample size, lack of large-scale multicenter validation, and untested applicability in real-world settings. |
| Ian Morilla  [18] | 2019 | Not reported | Not reported | Unsupervised feature selection, false discovery rate control, and linear programming | 9 microRNAs and 5 clinical parameters (14 total features) | Deep neural network | Leave-one-out and K-fold cross-validation | AUC: 0.91 (external validation) | Not reported | Not reported | Not reported | Not reported | Limitations include the retrospective study design, small sample size, lack of prospective validation, and limited generalizability due to the use of formalin-fixed paraffin-embedded tissue samples. |
| Ian Morilla [19] | 2021 | Not reported | Not reported | Bayesian inference, generalized linear models, and literature-based feature selection | 11 microRNAs (miRNAs) and 3 clinical factors (including ileitis, extraintestinal manifestations, and platelet count) | Random forest and linear regression combined | Not reported | AUC: 0.90 (external validation) | Sensitivity: 0.82; Specificity: 0.91 (external validation) | Not reported | Not reported | Not reported | Limitations include the retrospective study design, small sample size, unclear criteria for control group selection, and lack of validation for predicting acute pouchitis. |
| Tetsuro Takayama [20] | 2015 | Not reported | Not reported | Leave-one-feature-out analysis combined with artificial neural network modeling | Age, gender, lesion extent, disease duration, colonoscopy-associated phenotype type, clinical activity index score, and treatment history (eg, prednisolone, 6-mercaptopurine/azathioprine) | Artificial neural network | Random data split with repeated 4-fold cross-validation | Not reported | Sensitivity: 0.96; Specificity: 0.97 (external validation) | Not reported | Not reported | Not reported | Limitations include the poor interpretability of the artificial neural network model, potential overfitting, bias due to the retrospective study design, and the lack of external validation data. |
| Zhijun Bu [21] | 2023 | Direct Deletion | Randomly split into training and testing sets in a 70:30 ratio | Least absolute shrinkage and selection operator (LASSO) regression | Erythrocyte sedimentation rate (ESR), age, disease type, presence of microscopic bleeding, presence of pus, lesion location, and presence of pain | Logistic regression | Not reported | AUC: 0.699 (internal validation) | Not reported | Calibration curve was provided but showed poor performance | Not reported | Developed for remission evaluation in patients with active UC; an online calculator is available at https://  bzj0416.shinyap ps.io/  DynNomapp/ | Limitations include poor calibration performance, reliance on data from a single randomized controlled trial, and lack of external validation. |
| Na Li [22] | 2022 | Not reported | Randomly split into training and validation sets in a 70:30 ratio | Univariate and multivariate Cox proportional hazards models with stepwise backward selection | Age >43 years, moderate-to-severe disease activity, CRP >1.32 mg/L, and platelet count >240×10⁹/L | Cox regression | Bootstrap validation with 1000 iterations | AUC: 0.782 (95% CI: 0.695‐0.868), C-index: 0.759 (internal validation) | Not reported | Calibration curve showed good agreement in the training set, with slight deviations observed in the validation set | Not reported | Nomogram was provided for clinical application | Limitations include the retrospective study design, single-center setting, moderate model performance, and lack of integration with multi-omics data |
| Cong Dai [23] | 2024 | Not reported | Randomly split into training and validation sets in a 70:30 ratio | Univariate and multivariate logistic regression | Disease classification, monocyte count, platelet distribution width, total cholesterol level, and α₁-globulin concentration | Logistic regression | Bootstrap validation with 1000 iterations (internal validation) | AUC: 0.852 (internal validation) | Sensitivity: 0.793; Specificity: 0.802 (internal validation) | Calibration curve showed good agreement between predicted and observed values | Decision curve analysis (DCA)^g^ indicated high net clinical benefit within a predicted probability range of 0% to 96% | Nomogram was provided for clinical application | Limitations include the retrospective study design, moderate model performance, lack of external validation, and methodological limitations in endoscopic evaluation |
| Jie Chen [24] | 2023 | Direct deletion (complete-case analysis) | Randomly split into training and validation sets in a 2:1 ratio | Logistic regression with stepwise forward selection | Lesion extent, endoscopic findings, histological changes (Nancy Index), initial treatment drug, and relapse frequency | Logistic regression | Bootstrap validation to reduce the risk of overfitting | AUC: 0.925 (95% CI: 0.865‐0.984, external validation) | Sensitivity: 0.879; Specificity: 0.85 (external validation) | Calibration curve showed good fit in the training set, with slightly lower but acceptable fit in the validation set | Not reported | Developed to identify high-risk patients with acute severe ulcerative colitis (ASUC)^i^ resistant to intravenous corticosteroids; a nomogram was provided | Limitations include the retrospective study design, small validation cohort size, and exclusion of biomarker data |
| Si Yu [25] | 2022 | Not reported | Randomly split into training and validation sets in a 70:30 ratio | Lasso Regression | CRP level on day 3 and UCEIS score | Logistic regression | 100 iterations of random-split cross-validation | AUC: 0.873 (95% CI: 0.704‐1.000, internal validation); 0.703 (95% CI: 0.473‐0.934, external validation) | Not reported | Calibration curve for the logistic regression model indicated good fit | DCA indicated good clinical utility of the logistic regression model | Nomogram was provided for clinical application | Limitations include the small sample size, single-center study design, and calibration bias observed in the external validation cohort |
| Gi-Ung Kang [26] | 2022 | Not reported | Not reported | LASSO Regression | Key gut microbiota genera: Enterococcus, Rothia, and Colidextribacter | Logistic regression | 5-fold cross-validation | AUC: 0.844, internal validation | Not reported | Not reported | Not reported | Not reported | Limitations include small sample size, lack of external validation, and absence of long-term follow-up data |
| Akbar K. Waljee [27] | 2018 | Direct deletion (complete-case analysis) | Randomly split into training and validation sets in a 70:30 ratio | Random Forest | Fecal calprotectin, serum albumin, neutrophil count, white blood cell count, Vedolizumab concentration, and Vedolizumab concentration slope | Random forest | 50 iterations of random-split validation (no bootstrap) | AUC: 0.73 (95% CI: 0.65‐0.82, internal validation) | Sensitivity: 0.72; Specificity: 0.68 (internal validation) | Not reported | Not reported | Not reported | Limitations include the use of data derived from clinical trials, lack of external validation, and potential bias due to the absence of corticosteroid use at baseline |
| Jun Miyoshi [28] | 2021 | Mean imputation | Not reported | Embedded feature selection using random forest | Partial Mayo score, mean corpuscular hemoglobin, body mass index, blood urea nitrogen, azathioprine use, lymphocyte ratio, height, and CRP | Logistic regression | Cross-validation using random forest for hyperparameter tuning | Not reported | Not reported | Not reported | Not reported | Not reported | Limitations include small sample size, data limited to a Japanese population, retrospective study design, and lack of comparative validation with alternative therapies |
| Hiromu Morikubo [29] | 2024 | Mean imputation or mode imputation | Not reported | Embedded feature selection using random forest | Serum albumin, monocyte count, height, mean corpuscular volume, total protein, Lichtiger index, white blood cell count, mean corpuscular hemoglobin concentration, and CRP | Support vector machine | 5-fold cross-validation | AUC: 0.673 (external validation) | Sensitivity: 0.846; Specificity: 0.50 (external validation) | Not reported | Not reported | Not reported | Limitations include small sample size, potential bias from retrospective data, and limited external validation |
| Susan D. Ghiassian [30] | 2022 | Not reported | Not reported | Feature selection using Pearson correlation, signal-to-noise ratio, and human interactome network analysis | Twelve selected genes (eg, AMIGO2, CXCL1, MMP12) | Probabilistic neural network | Leave-one-subject-out cross-validation (LOSO-CV) | AUC: 0.83 (external validation) | Sensitivity: 0.64 (external validation) | Not reported | Not reported | Not reported | Limitations include small sample size, potential bias in genetic data, and lack of prospective validation |
| Sofo [31] | 2020 | Not reported | Not reported | Feature extraction and normalization | Age, gender, treatment history (eg, steroid use), hemoglobin, white blood cell count, serum albumin, lymphocyte count, serum creatinine, surgical approach, blood transfusion, hospital stay duration, and body temperature | Support vector machine | Leave-one-out cross-validation | Not reported | Sensitivity: 0.875; Specificity: 0.833 (internal validation) | Not reported | Not reported | Not reported | Limitations include small sample size, low incidence of complication events, potential bias from retrospective design, and insufficient model optimization |
| Jing Feng [32] | 2021 | Not reported | Not reported | Differential expression analysis using Limma and feature selection via random forest | Thirty selected genes (eg, IL13RA2, TNFRSF11B, CXCL8) | Artificial neural network | Not reported | AUC: 0.81 (external validation) | Not reported | Not reported | Not reported | An online calculator is available via Streamlit at https://uc2023.streamlit.app | Limitations include small sample size, lack of experimental validation, and applicability limited to predicting non-response to infliximab in patients with UC |
| Tom Konikoff [33] | 2024 | Not reported | Randomly split into training and validation sets in a 90:10 ratio | Embedded feature selection using CatBoost | Age, serum phosphate levels, CRP levels, platelet count, and hemoglobin (Hb) levels | CatBoost | Not reported | AUC: 0.86 (internal validation) | Sensitivity: 0.888 (internal validation), 0.888 (external validation); Specificity: 0.5 (internal validation), 0.272 (external validation) | Provided calibration curve; good consistency between predicted probabilities and observed values | Not reported | A scoring model (0‐3 points) and an individualized risk nomogram were provided | Limitations include data restricted to a single country, missing data for some variables, and lack of long-term follow-up |
| Monica Cesarini [34] | 2017 | Multiple imputation | Not reported | Predictor selection based on literature review and multivariate logistic regression | Lesion extent (E3 vs E1/E2), CRP >10 mg/L, hemoglobin levels (males <14 g/dL, females <12 g/dL) | Logistic regression | Bootstrap internal validation (200 resamples) | Concordance index (C-index)^j^: 0.97 (external validation) | Sensitivity: Not reported (internal validation), 0.92 (external validation) | Calibration curve showed good agreement between predicted risks and observed outcomes (internal and external validation) | Not reported | A nomogram was provided for clinical use | Limitations include retrospective design, cohort variability, lack of treatment data collection, and the need to validate predictor thresholds |
| Mohammad Hossein Derakhshan Nazari [35] | 2023 | Not reported | Not reported | LASSO regression | Five mRNA biomarkers (IL13RA2, HCAR3, CSF3, INHBA, and MMP1) | Two-layer stacked ensemble machine learning model | Ten repeated 10-fold cross-validations | AUC: 0.981 (internal validation), 0.948 (external validation) | Sensitivity: 1.000 (internal validation), 0.890 (external validation); Specificity: 0.880 (internal), 0.980 (external validation) | Not reported | Not reported | Not reported | Limitations include small sample size, use of public datasets from a single center, and lack of validation across multiple anti-TNF agents |
| Seok-Young Kim [36] | 2024 | Not reported | Not reported | Not reported | All 7201 wavelength features from spectral measurements | Logistic regression | 10-fold cross-validation | AUC: 0.990 (internal validation, 95% CI: 0.970‐1.010), 0.750 (external validation) | Sensitivity: 0.980 (internal validation), 0.730 (external validation); Specificity: 0.720 (external validation) | Not reported | Not reported | Not reported | Limitations include small sample size, data limited to a single country, lack of incorporation of other biomarkers, and the need for large-scale validation |
| Charlie W. Lees [37] | 2021 | Direct deletion of missing data | Not reported | Forward stepwise selection | Mayo score, cholesterol levels, CRP levels, and their temporal trends | Logistic regression | 10-fold cross-validation with repeated validation | AUC: 0.870 (internal validation) | Not reported | Not reported | Not reported | Not reported | Limitations include data derived from clinical trials, variability in subjective variables, and the need to validate the clinical significance of laboratory indicators |
| Uday C. Ghoshal [38] | 2020 | Not reported | Not reported | Univariate analysis followed by principal component analysis | Age, gender, hemoglobin, total leukocyte count, neutrophil percentage, platelet count, albumin, potassium, ESR, CRP, complications, hospital stay duration, prothrombin time | Artificial neural network | Not reported | Not reported | Not reported | Not reported | Not reported | Not reported | Limitations include a small number of non-responders, single-center retrospective design, and lack of external validation |
| Mizuno [39] | 2022 | Not reported | Not reported | Deep feature extraction using convolutional neural network | Deep learning features extracted from endoscopic images | Convolutional neural network | 5-fold cross-validation | AUC: 0.840 (internal validation) | Not reported | Calibration curve showed good agreement between predicted probabilities and observed outcomes | Not reported | Not reported | Limitations include single-center study, small sample size, variability in endoscopic procedures, and potential bias from retrospective design |
| Wenwen Pang [40] | 2023 | Not reported | Randomly split into training set (70%), 30% test set | Univariate regression followed by stepwise regression | Age, gender, routine blood test parameters, serological markers, and informationrelated indices (eg, ESR, D-dimer) | Random forest | Bootstrap validation | AUC: 0.889 (internal validation) | Sensitivity: 0.785，Specificity: 0.764 (internal validation) | Calibration curve showed good agreement between predicted risks and actual outcomes | DCA indicated high net benefit across a range of threshold probabilities | Nomogram was provided for clinical application | Limitations include conservative design, small sample size, retrospective design, and exclusion of potential predictors |
| Jingjing Chen [41] | 2022 | Direct deletion of missing data | 3:1 split (training:validation) | Elastic net regularized regression, two-stage training | Demographics, disease characteristics, medication history, and treatment technique | Elastic net regularized regression | 5-fold cross-validation repeated 100 times | AUC: 0.811 (internal validation) | Sensitivity: 0.474 (internal validation); Specificity: 0.868 (internal validation) | Not reported | Not reported | Not reported | Limitations include reliance on randomized controlled trial data, limited generalizability to real-world settings, exclusion of dynamic variables, and poor interpretability of the random forest model |
| Zhongyuan Wang [42] | 2023 | Not reported | Not reported | Univariate analysis followed by multivariate logistic regression | Preoperative anti–tumor necrosis factor-- α treatment, eosinophil percentage>1.45 %, and procalcitonin>0 . 0635 μg/L | Logistic regression | 5-fold cross- validation repeated 100 times | C-index: 0.747 (95% CI: 0.656-0.893, internal validation) | Not reported | Calibration curve demonstrated acceptable calibration, roughly aligned with the reference line | Not reported | Nomogram was provided for clinical application | Limitations include single-center retrospective design, small sample size, and need for multicenter validation |
| Xuehui Wang [43] | 2023 | Direct deletion of missing data | Not reported | Univariate analysis followed by multivariate logistic regression | Age, disease type, absence of microscopic bleeding, reduction in ESR | Logistic regression | Bootstrap validation (1000 times) | C-index: 0.735 (95% CI: 0.672- 0.797, internal validation); 0.540 (95% CI: 0.440-0.650, external validation) | Not reported | Calibration curve assessed good consistency between predicted risk and actual outcome, and minimum failure rates | Not reported | Model selection: Nomogram provided | Limitations include small external validation sample size, low model likelihood index (1.58%), and reliance on randomized data, which limits applicability to real-world settings |

^a^AIC=Akaike Information Criterion

^b^CRP=C-reactive protein

^c^AUC=Area under the curve

^d^CI=Confidence interval

^e^UC=Ulcerative colitis

^f^KNN=k-nearest neighbors

^g^DCA=Decision curve analysis

^h^UCEIS=UC Endoscopic Index of Severity

^i^ASUC=Acute severe ulcerative colitis

^j^C-index=Concordance index
