# Supplementary material for: Prognostic Prediction Models for Ulcerative Colitis: Systematic Review and Meta-Analysis
Source: J Med Internet Res. 2025 Dec 22;27:e71944. doi: 10.2196/71944 (PMC12721486; doi:10.2196/71944)
Supplement: Multimedia Appendix 4 [file jmir-v27-e71944-s004.docx]

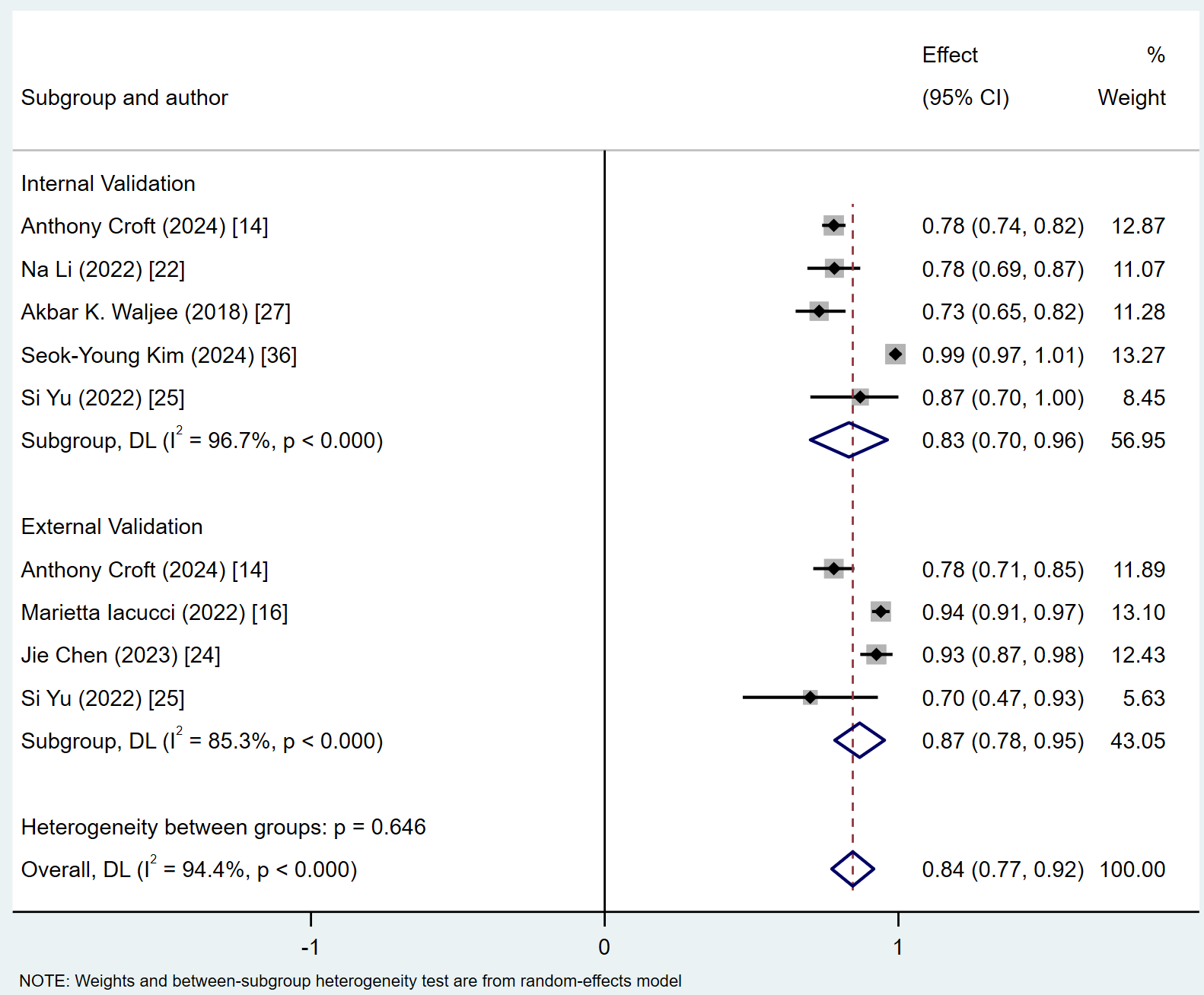


**Figure S1.** Forest plot of pooled area under the curve (AUC) values for prognostic prediction models in ulcerative colitis (UC), stratified by validation type. This meta-analysis summarizes the diagnostic performance of prognostic models for UC based on studies published in either English or Chinese, from the inception of each database up to November 2, 2024. The included studies were conducted across diverse clinical settings and geographic regions. Each square represents the AUC of an individual study with 95% CIs, whereas diamonds indicate the pooled AUC estimates for internal validation, external validation, and overall, calculated using a random-effects model. Between-study heterogeneity was assessed using the I² statistic and corresponding P values.

**Figure S2.** Funnel plot for area under the curve (AUC) values in internal validation. Each dot represents a study included in the meta-analysis. The x-axis shows the effect size (AUC), and the y-axis represents the standard error of the effect size. The vertical line indicates the pooled effect estimate, and the dashed lines represent the pseudo 95% confidence limits. The asymmetry of the plot suggests the presence of potential publication bias in the included studies.

**Figure S3.** Egger’s regression intercept plot evaluating publication bias based on AUC values derived from internal validation. The x-axis represents the precision (inverse of the standard error), and the y-axis shows the standardized effect size. Each point corresponds to an individual study included in the meta-analysis. The regression line tests whether the intercept significantly differs from zero, which would indicate asymmetry in the funnel plot and potential publication bias. The observed distribution supports the presence of small-study effects.

**Figure S4.** Sensitivity analysis for internal validation AUC values. The plot shows the influence of removing each individual study on the overall pooled estimate in a leave-one-out meta-analysis. Each horizontal line represents the 95% confidence interval (CI) of the pooled effect size when the corresponding study is omitted. The consistency of the pooled estimate across iterations suggests that no single study excessively influenced the overall results.


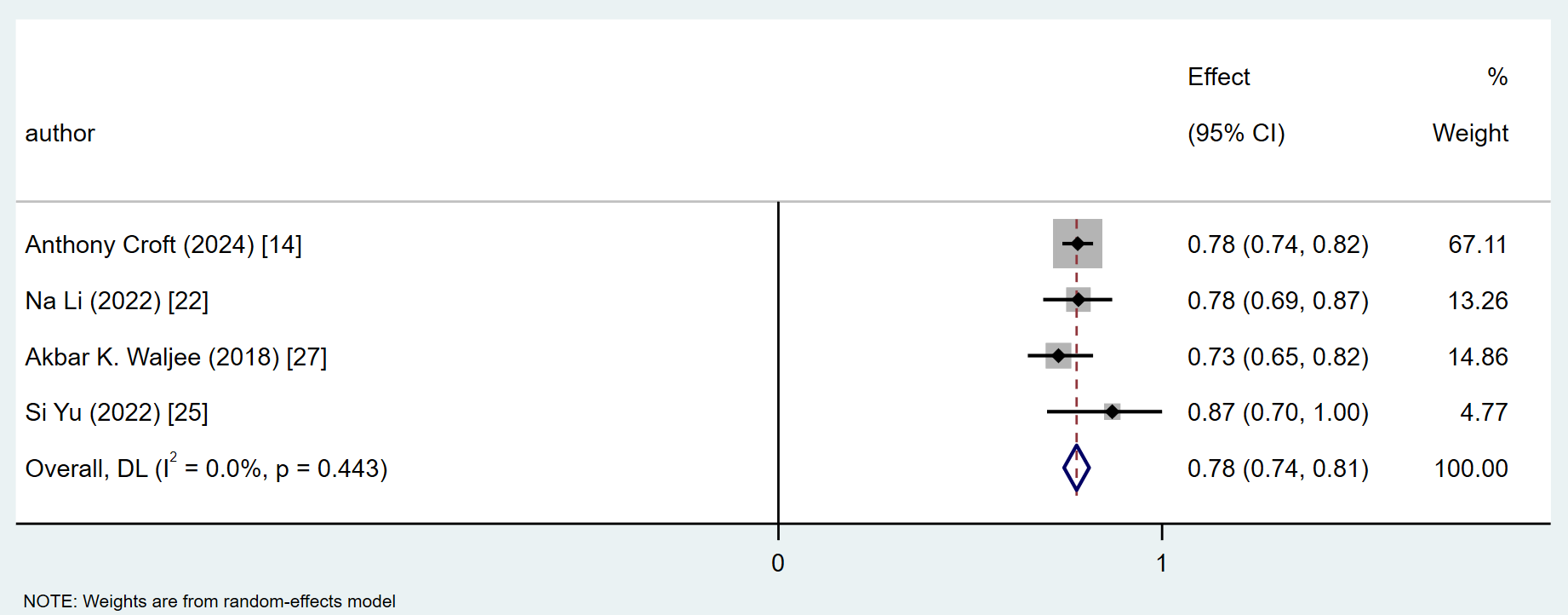


**Figure S5.** Forest plot of AUC values for internal validation after excluding the study by Seok-Young Kim (2024). The plot presents the updated pooled effect size and heterogeneity results. The x-axis shows the AUC values with 95% confidence intervals (CIs), and the diamond represents the overall pooled estimate from a random-effects model. The reduced I² value (0, p = 0.443) indicates decreased heterogeneity, suggesting that the excluded study may have contributed to variability among the included studies.
